# Yeast‐Raised Polyamidoxime Hydrogel Prepared by Ice Crystal Dispersion for Efficient Uranium Extraction from Seawater

**DOI:** 10.1002/advs.202306534

**Published:** 2024-02-13

**Authors:** Hui Wang, Weikun Yao, Yihui Yuan, Se Shi, Tao Liu, Ning Wang

**Affiliations:** ^1^ State Key Laboratory of Marine Resource Utilization in South China Sea Hainan University Haikou 570228 China

**Keywords:** ice crystal dispersion, macroporous hydrogels, polyamidoxime, uranium extraction, yeast‐based biological foaming

## Abstract

Uranium extraction from seawater has attracted worldwide attention due to the massive reserves of uranium. Due to the straightforward synthesis and strong affinity toward uranyl ions (UO_2_^2+^), the amidoxime group shows promise for use in highly efficient uranium capture.  However, the low mass transfer efficiency within traditional amidoxime‐based adsorbents severely limits the adsorption rate and the utilization of adsorption sites. In this work, a macroporous polyamidoxime (PAO) hydrogel is prepared by yeast‐based biological foaming combined with ice crystal dispersion that effectively maintained the yeast activity. The yeast‐raised PAO (Y‐PAO) adsorbent has numerous bubble‐like holes with an average pore diameter >100 µm. These macropores connected with the intrinsic micropores of PAO to construct efficient diffusion channels for UO_2_^2+^ provided fast mass transporting channels, leading to the sufficient exposure of hidden binding sites. The maximum adsorption capacity of Y‐PAO membrane reached 10.07 mg‐U/g‐ads, ≈1.54 times higher than that of the control sample. It took only eight days for Y‐PAO to reach the saturation adsorption capacity of the control PAO (6.47 mg‐U/g‐ads, 28 days). Meanwhile, Y‐PAO possessed excellent ion selectivity, good reusability, and low cost. Overall, the Y‐PAO membrane is a highly promising adsorbent for use in industrial‐scale uranium extraction from seawater.

## Graphical abstract

In this work, a macroporous polyamidoxime (PAO) hydrogel is prepared by yeast‐based biological foaming combined with ice crystal dispersion that effectively maintained the yeast activity. The yeast‐raised PAO (Y‐PAO) adsorbent has numerous bubble‐like holes with an average pore diameter >100 µm. Overall, the Y‐PAO membrane is a highly promising adsorbent for use in industrial‐scale uranium extraction from seawater.

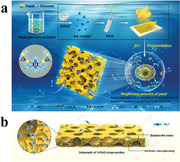

## 1 Introduction

To achieve the goal of carbon neutrality, technologies related to clean energy development, low‐loss energy transmission, and high energy density storage have gained considerable attention from researchers.^[^ ^1^ ^]^ As an efficient and low emission source of electricity, nuclear power functions as a major pillar for reducing human dependence on fossil fuels and combating climate change.^[^ ^2^ ^]^ Uranium is a crucial resource in the nuclear energy industry, and it is mainly sourced from the traditional uranium ore (U_3_O_8_).^[^ ^3^ ^]^ However, the current available terrestrial uranium mines, which are sparsely distributed across the world, may be depleted in less than a century.^[^ ^4^ ^]^ Owing to the increasing demand for uranium and the gradual exhaustion of mineral resources, an alternative way to acquire uranium resources is urgently needed. Seawater contains roughly 4.5 billion tons of uranium, which is 1000 times higher than that on land. Thus, the uranium resource in seawater could indefinitely serve nuclear power plants to satisfy the human production^[^ ^5^ ^]^ and living needs.^[^ ^6^ ^]^ Therefore, the extraction of uranium from natural seawater is of great importance for the sustainable development of nuclear power production.

Uranium in seawater exists in the form of a tricarbonate complex ([UO_2_(CO_3_)_3_]^4−^) with an extremely low concentration of ≈3.3 ppb.^[^ ^7^ ^]^ Moreover, uranium extraction from natural seawater suffers from numerous competing ions, biological fouling, and harsh marine environments.^[^ ^8^ ^]^ In response to these difficulties, different methods, including ion exchange,^[^ ^9^ ^]^ adsorption,^[^ ^10^ ^]^ chemical precipitation,^[^ ^11^ ^]^ and biological capture,^[^ ^12^ ^]^ have been developed to realize marine uranium uptake. Among them, adsorption has been identified as the most promising approach for industrial application in terms of economic feasibility and simple operation.^[^ ^13^ ^]^ In particular, amidoxime‐based adsorbents, which can be directly synthesized from nitrile‐containing materials by an oximation reaction, have received widespread attention owing to the excellent affinity toward uranyl ions (UO_2_^2+^) in high‐salt natural seawater.^[^ ^14^ ^]^ Diverse amidoxime‐based adsorbents, such as fibers,^[^ ^15^ ^]^ aerogels,^[^ ^16^ ^]^ hydrogels,^[^ ^17^ ^]^ and chelating resins,^[^ ^18^ ^]^ have been prepared for effective uranium capture.^[^ ^19^ ^]^ Notably, polyamidoxime (PAO) hydrogel adsorbents constructed by chemical and/or physical crosslinking exhibit good hydrophilicity, have a porous structure, and can be easily recovered, all of which are advantageous features.^[^ ^20^ ^]^ However, the 3D crosslinked network of the hydrogel can trap abundant water molecules by forming hydrogen bonds. This situation not only limits the diffusion of UO_2_^2+^ in the hydrogel,^[^ ^21^ ^]^ but also prevents many of the active binding sites from being fully utilized.^[^ ^22^ ^]^ In addition, a long soak in seawater inevitably subjects the adsorbents to biological fouling, further decreasing the adsorption capacity. To enhance the adsorption capacity and adsorbent rate of the PAO hydrogel, ultrafast mass transporting channels were designed and synthesized in a previous study. For example, a macroporous PAO hydrogel was prepared using a physical pore‐forming agent (NaHCO_3_)_._ The improved diffusion of UO_2_^2+^ contributed to the increase in the adsorption capacity in natural seawater.^[^ ^23^ ^]^ Owing to the limited number of macropores, however, the uranium adsorption capacity remained low. Vertically aligned PAO‐graphene oxide films were successfully prepared by directional freeze‐casting. The penetrating microchannels significantly accelerated the free diffusion of UO_2_^2+^, leading to a high adsorption capacity (13.63 ± 0.24 mg‐U/g‐Ads) and fast adsorption rate (0.43 mg g^−1^ day^−1^) in natural seawater.^[^ ^24^ ^]^ Inspired by the transpiration mechanism of plants, a plant‐mimicking directed channel PAO (DC‐PAO) hydrogel was prepared to enhance the uranium extraction efficiency by actively pumping UO_2_^2+^ into the adsorbent. Compared to that of the pristine PAO hydrogel, the uranium extraction capacity of the DC‐PAO hydrogel increased by 79.33% in natural seawater.^[^ ^25^ ^]^ However, considering the complex preparation process and the high economic costs, however, these adsorbents are difficult to apply for practical industrialization.

Yeasts have been extensively used by people to prepare fluffy bread and biscuits.^[^ ^26^ ^]^ Inspired by steamed bread dough fermentation, yeast can serve as a foaming agent by releasing a substantial amount of gases in materials.^[^ ^27^ ^]^ Recently, yeasts have also been widely utilized in other applications, such as porous Al_2_TiO_5_–mullite ceramics, alumina honeycomb ceramics,^[^ ^28^ ^]^ and wearable biosensors.^[^ ^29^ ^]^ Active dry yeast is inexpensive and easy to obtain and store.^[^ ^30^ ^]^ The fermentation process only required simple equipment and consumed less reaction energy.^[^ ^31^ ^]^ However, yeast, as an organism, has specific requirements for the environment in which it lives. A weakly acidic comfortable environment with suitable moisture and temperature is necessary for the sufficient respiration of yeast. Furthermore, yeast has a low tolerance for organic solvents, which affect the integrity of the cell membrane and thus lead to cell inactivation.^[^ ^32^ ^]^ The wide application of yeast is blocked by the above‐mentioned difficulties.

In this study, PAO was first prepared by the oximation reaction of polyacrylonitrile (PAN) in dimethylformamide (DMF), where DMF is the indispensable organic solvent for the synthesis of PAO. Then, a yeast‐raised PAO (Y‐PAO) hydrogel adsorbent with abundant bubble‐like pores could be synthesized by virtue of yeast‐based biological foaming. In order to maintain the activity of yeast in DMF organic solvents, the living yeast was encapsulated into ice crystals and then evenly dispersed in DMF solvent containing PAO (PAO/DMF). The large pores facilitated mass diffusion into the adsorbent, which enabled more amidoxime groups to bind with UO_2_^2+^. Consequently, the adsorption capacity and adsorption rate of Y‐PAO membrane were significantly improved compared with those of the control PAO without yeast. The whole preparation process is simple, low‐cost, and easy to scale up. The ice crystal dispersion approach could also be used in other situations to maintain the activity of organisms in organic solvents, which may be considered as a promising strategy for the extended application of biological ferment.

## 2 Results and Discussion

### 2.1 Preparation and Characterization of Y‐PAO Membranes

**Figure 1:**
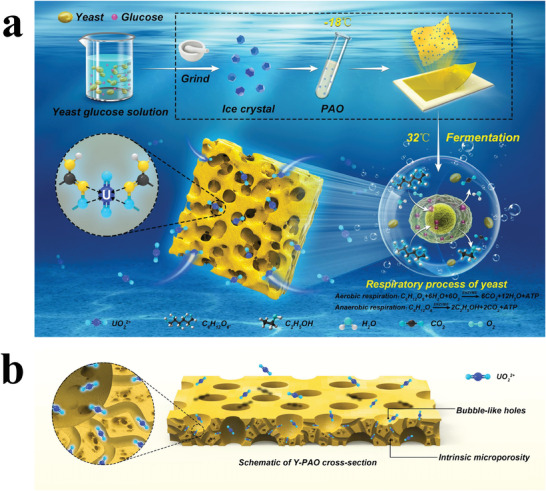
Yeast‐raised macroporous Y‐PAO adsorbents. a) Schematic illustration of the preparation of Y‐PAO by combination of ice crystal dispersion and yeast fermentation. b) Diagram of fast ion diffusion channels constructed by the hierarchical porous structure including large bubble‐like pores and the intrinsic micropores of Y‐PAO.

The preparation process of the Y‐PAO membrane is depicted in **Figure** **1**a. First, a certain amount of yeast was mixed into an aqueous glucose solution, which served as a reaction energy source for yeast fermentation. Subsequently, the mixture was frozen at −18 °C and ground into ice crystal particles. This process helps preserve the activity of yeast in an organic solvent. Afterward, the as‐prepared ice crystal particles were poured into PAO/DMF solvent at −18 °C. DMF could be removed by solvent extraction at a low temperature. Finally, the product was heated to temperatures suitable for yeast respiration (≈32–35 °C).^[^ ^33^ ^]^ Consequently, the abundant bubble‐like pores were generated in the Y‐PAO hydrogel via yeast respiration. The hierarchical porous structure containing large bubble‐like pores and the intrinsic micropores constructed efficient diffusion channels for UO_2_^2+^ (Figure 1b).

**Figure 2:**
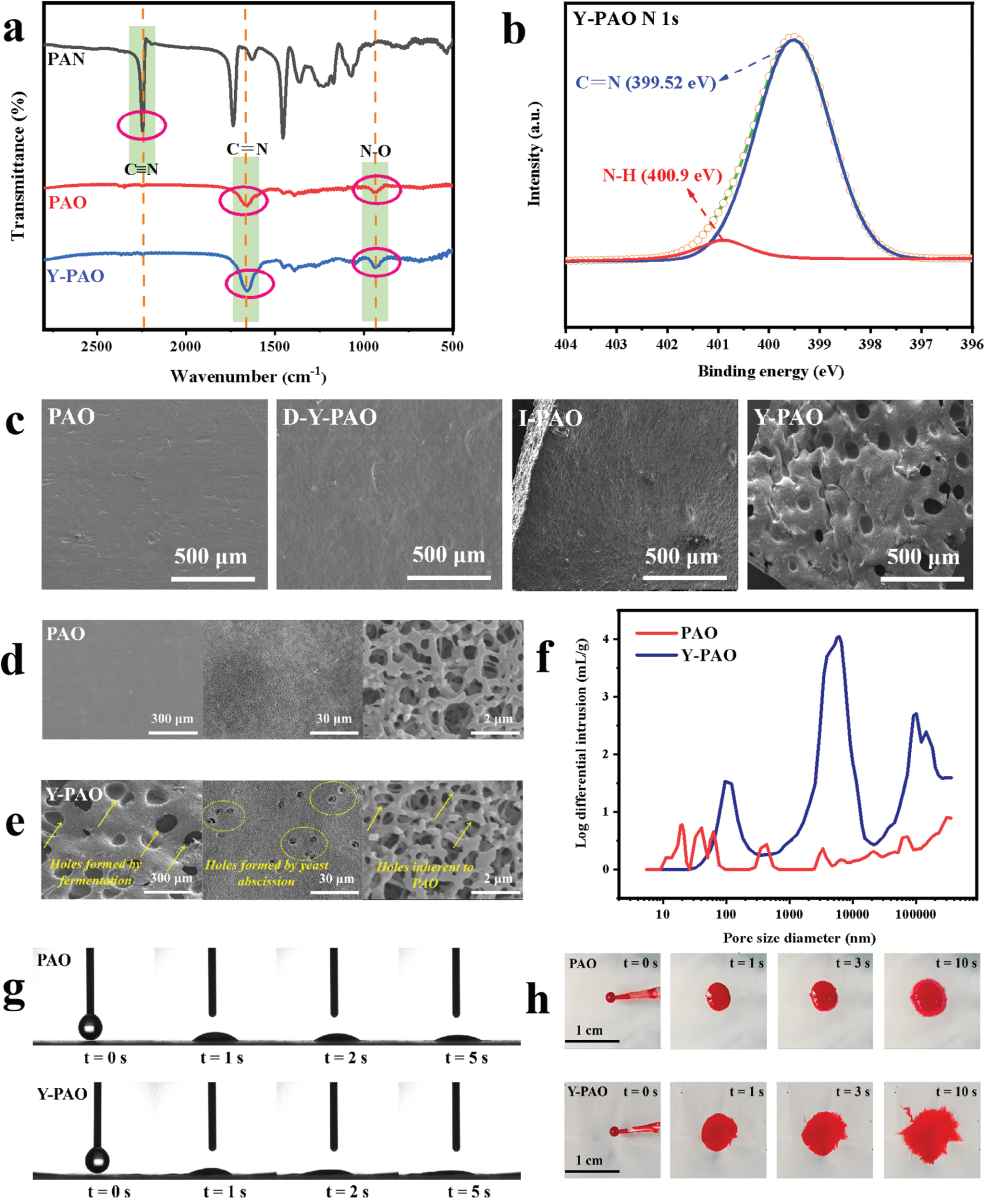
Composition, structure and characterization. a) FTIR spectra of PAN, PAO, and Y‐PAO. b) High‐resolution N 1s XPS spectrum of the Y‐PAO membrane. c) SEM images of PAO, D‐Y‐PAO, I‐PAO, and Y‐PAO. SEM images of d) PAO and e) Y‐PAO membrane surfaces at different magnifications. f) MIP analysis of the PAO membrane and Y‐PAO membrane. g) Water contact angle tests of PAO and Y‐PAO. h) Permeability test results of PAO and Y‐PAO membranes. The liquid is the red ink.

PAN, PAO, and Y‐PAO were analyzed by Fourier‐transform infrared (FTIR) spectroscopy (**Figure** **2**a). C≡N stretching vibration (2250 cm^−1^) is observed in the FTIR spectrum of PAN. PAO and Y‐PAO exhibit two characteristic peaks ascribed to N─O (945 cm^−1^) and C═N (1685 cm^−1^), whereas the peak representing C≡N band disappears. This finding demonstrates the successful transformation of the PAN nitrile group into an amidoxime moiety. The high‐resolution N1s X‐ray photoelectron spectroscopy (XPS) spectrum of Y‐PAO shows two peaks ascribed to C═N and N─H, which further verify the appearance of the amidoxime group in PAO (Figure 2b). The high‐resolution C1s spectrum of Y‐PAO presents four deconvoluted peaks (Figure S1, Supporting Information), which can be ascribed to C─C, C═N, C═O, and O─C═O bonds. This result also confirms the successful transformation of C≡N into C═N.

Next, we provide an in‐depth look at the ice crystal dispersion method to maintain the respiration of yeast in DMF organic solvent. For comparison, yeast solution and ice crystals were directly mixed with PAO/DMF to prepare D‐Y‐PAO and I‐PAO, respectively. Figure 2c shows the scanning electron microscope (SEM) images of the control PAO, D‐Y‐PAO, I‐PAO, and Y‐PAO. The surfaces of the control PAO and D‐Y‐PAO samples are smooth, whereas the surface of I‐PAO is a little rougher than that of PAO owing to the occupation of ice crystals. However, no bubble‐like pores were observed on the control samples. By contrast, there are large numbers of bubbles with an average diameter of ≈100 µm on the surface of Y‐PAO, which fully proves that the ice crystal dispersion method can effectively preserve the biological activity of yeast, thereby allowing the full respiration of yeast. The yeast fermentation in Y‐PAO causes the adsorbent to become loose and porous. The microstructures of the control PAO and Y‐PAO membrane were further investigated by SEM imaging (Figures 2d,e). The pore sizes of Y‐PAO are mainly approximately 100 nm, 5 µm, and 100 µm, indicating a hierarchical porous structure of Y‐PAO. By contrast, only ≈100‐nm‐sized micropores appear in the control PAO membranes. The yeast has a uniform diameter of approximately 5 µm (Figure S2, Supporting Information). The pore morphology of the Y‐PAO membrane reveals bubble‐like pores generated by yeast respiration (scale bar, 300 µm), ≈5‐µm‐sized pores produced via yeast abscission (scale bar, 30 µm), and the nanoscale pores inherent in the PAO membrane (scale bar, 2 µm), as further supported by mercury intrusion porosimetry (MIP) analysis (Figure 2f). The pore volume of Y‐PAO membrane is almost four times larger than that of the PAO membrane (Figure S3, Supporting Information). Compared to the control PAO, Y‐PAO has a large number of internal pores, which can significantly enhance the diffusion of UO_2_^2+^ during the adsorption process.

Water contact angle tests (Figure 2g) indicated that the Y‐PAO membrane exhibited superior hydrophilicity compared to the control PAO membrane. In addition, a water diffusion test was performed to evaluate the permeability of the PAO and Y‐PAO membranes (Figure 2h). In this experiment, 2.5 µL of red ink was added dropwise onto the membrane surfaces. Due to the fast mass diffusion channels constructed by hierarchical porous structure, the ink traces on Y‐PAO spread radially toward the edges quickly. Obviously, the ink diffusion area on the Y‐PAO membranes is larger than that on the PAO membranes. Owing to the existence of numerous holes, the density of the Y‐PAO membranes is significantly lower than that of the PAO membranes (Figure S5, Supporting Information). As shown in Figure S6 (Supporting Information), the tensile strength of the Y‐PAO hydrogel adsorbent increases with decreasing yeast content. Even so, the tensile strength remains higher than 1 MPa when the value of V_PAO_/V_Yeast_ is greater than 10, indicating that the Y‐PAO membrane can exhibit good mechanical properties for withstanding external impacts in the marine environment.

### 2.2 Uranium Extraction Performance from Simulated Seawater

**Figure 3:**
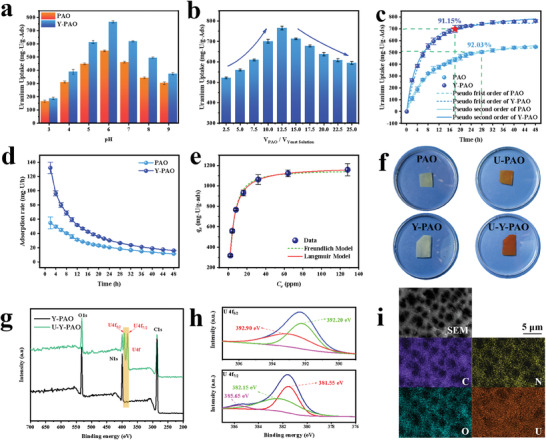
Uranium adsorption performance of Y‐PAO in 8 ppm uranium‐spiked simulated seawater (*V*, 1000 mL; *M*_ads_, 5 mg). a) pH‐dependent uranium adsorption capacities of PAO and Y‐PAO. b) Effect of yeast contents on uranium adsorption capacity of Y‐PAO membrane. c) Uranium adsorption kinetic data and corresponding fitting curves based on pseudo‐first‐order and pseudo‐second‐order models for PAO and Y‐PAO. d) Uranium adsorption rates of PAO and Y‐PAO. e) Equilibrium adsorption isotherm and corresponding fitting curves based on Freundlich model and Langmuir model for Y‐PAO membrane. f) Digital photographs of the PAO and Y‐PAO membranes before and after uranium adsorption. g) XPS spectra of Y‐PAO before and after uranium adsorption. h) High‐resolution U4f XPS spectra of the Y‐PAO membrane. (i) EDS mapping data of Y‐PAO after uranium adsorption.

It is well known that hydrothermal alkaline treatment can significantly improve the adsorption performance of amidoxime‐based adsorbents.^[^ ^34^ ^]^ As shown in Figure S7 (Supporting Information), ≈100‐µm‐thick PAO and Y‐PAO membranes weighing 5 mg were subjected to alkali treatment. Consequently, Y‐PAO exhibited a larger volume than that of the alkali‐treated PAO, indicating the superior swelling of Y‐PAO (Figure S8, Supporting Information). The alkali‐treated membranes were subsequently immersed in 1 L of uranium‐spiked simulated seawater to evaluate the uranium adsorption performance. The uranium adsorption performance can be affected by the pH value, which is closely related to the surface charge of the adsorbent and uranium species.^[^ ^35^ ^]^ **Figure** **3**a shows the pH‐value‐dependent uranium adsorption capacities of the PAO and Y‐PAO membranes. The uranium adsorption capacity clearly increases at first and then decreases as the pH increases from 3 to 9, and the maximum uranium extraction capacity is obtained at pH 6. Notably, the Y‐PAO membrane demonstrates a higher adsorption capacity than the PAO membrane regardless of the pH level, suggesting that the numerous bubble‐like holes in the Y‐PAO membrane contribute to the enhancement of the uranium uptake amount. Specially, the Y‐PAO membrane exhibits a high uranium adsorption capacity of 495.04 mg‐U/g‐Ads at pH 8, comparable to that of natural seawater (pH 8.3).

Y‐PAO membranes with different yeast contents were also prepared to investigate the effect of the macropore content on the uranium adsorption capacity. As shown in Figure 3b, the uranium adsorption capacity initially increases and then decreases with decreasing yeast. On the one hand, the increase in yeast results in a low amidoxime content in Y‐PAO, leading to decreased uranium adsorption capacity. On the other hand, more bubble pores were generated via fermentation with increasing yeast content (Figure S9, Supporting Information), resulting in a high adsorption capacity due to the favorable diffusion of UO_2_^2+^. Since PAO hinders the aggregation of bubbles, the size of the bubble‐liked pores does not change obviously with the yeast content. An increase in the yeast content will increases the number of bubbles rather than increase the size of the bubbles. The highest adsorption capacity of uranium was achieved when the solution ration of PAO/yeast was 12.5. Figure 3c shows the adsorption kinetics data of uranium in 8 ppm uranium‐spiked simulated seawater for PAO and Y‐PAO. The equilibrium adsorption capacity of uranium for the Y‐PAO membrane is 766.05 mg‐U/g‐Ads, which is 39.90% higher than that of the PAO membrane (547.58 mg‐U/g‐Ads). Moreover, the adsorption equilibrium of the Y‐PAO membrane can be achieved in a shorter time than that of the control PAO, as further verified by the time dependent adsorption rate (Figure 3d). The Y‐PAO membrane could reach 91.15% of the equilibrium adsorption capacity after 18 h, whereas it took a longer time (28 h) for the PAO membrane to reach 92.03% of the equilibrium adsorption capacity (Figure 3c). As listed in Table S1 (Supporting Information), the pseudo‐second‐order model had a higher correlation coefficient (*R* ^2^) than the pseudo‐first order model, indicating that the chemical adsorption was the rate‐limiting step. Moreover, the adsorption kinetics data of uranium at different initial concentrations (2, 4, and 16 ppm) are given in Figures S10–S12 (Supporting Information). The quasi‐first‐order and quasi‐second‐order fitting parameters are listed in Table S1 (Supporting Information). The uranium adsorption data of PAO and Y‐PAO at different times are provided in Table S2 (Supporting Information). Figure 3e shows the equilibrium adsorption isotherm of the Y‐PAO adsorbent. The Langmuir model better predicts the adsorption isotherm kinetic data (*R^2^* = 0.9983) compared to the Freundlich model (*R^2^* = 0.9899), suggesting that adsorption process is dominated by monolayer chemical adsorption. The maximum adsorption value can be as high as 1182.32 mg‐U/g‐Ads (Table S3, Supporting Information). Figure 3f shows digital photographs of PAO and Y‐PAO before and after uranium adsorption. Both membranes change from pale yellow to brown after adsorption, and the Y‐PAO membrane appears to be darker than PAO membrane. The coordination mechanism between Y‐PAO and UO_2_^2+^ was investigated by X‐ray photoelectron spectroscopy (XPS) and energy‐dispersive X‐ray spectroscopy (EDS). The U4f XPS spectrum of U‐Y‐PAO reveals double peaks centered at 392.3 and 381.5 eV (Figure 3g,h), which can be ascribed to U4f_5/2_ and U4f_7/2_, respectively, revealing successful uranium capture (Figure 3h). The EDS mapping data of U‐Y‐PAO (Figure 3i; Figure S13, Supporting Information) confirm the presence of abundant uranium in the membrane. As shown in Figure S14 (Supporting Information), the high‐resolution O1s core‐level spectra of Y‐PAO had two peaks obtained through Gaussian fitting, corresponding to N─O (532.5 eV) and C─O (530.9 eV). By contrast, U‐Y‐PAO exhibits a new peak centered at 531.5 eV, which was assigned to the O═U═O group, indicating the successful capture of UO_2_^2+^. Notably, the N‐O peak of U‐Y‐PAO shifted to a slightly lower binding energy than that of the N‐O peak of Y‐PAO, suggesting the sufficient coordination of UO_2_^2+^ with exposed terminal nitrogen and oxygen atoms from the ─NH_2_ and ─OH moieties of amidoxime, respectively. Similarly, the high‐resolution N1s core‐level spectra of U‐Y‐PAO exhibited a new peak centered at 401.45 eV, which could be attributed to the N‐U band (Figure S15, Supporting Information). The chemical coordination structure between the amidoxime groups and UO_2_^2+^ is illustrated in Figure S16 (Supporting Information).

**Figure 4:**
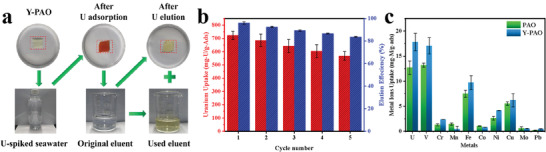
Reusability and uranium adsorption selectivity of Y‐PAO. a) Digital photographs of Y‐PAO used for cyclic adsorption–desorption tests. b) Cycle adsorption performance of Y‐PAO. c) Adsorption selectivity of PAO and Y‐PAO in simulated seawater.

The application reusability of Y‐PAO membrane was evaluated by adsorption–desorption cycling experiments (**Figure** **4**a). After five successive adsorption–desorption cycles, the uranium adsorption capacity and desorption efficiency remain high, reaching 568.26 mg‐U/g‐Ads and 83.74%, respectively (Figure 4b). The interference of completive metal ions (V, Cr, Mn, Fe, Ni, Co, Mo, Pb, and Cu) was also evaluated in this work (Figure 4c). Although obvious competition exists for the adsorption sites of Y‐PAO due to the similar charges and ionic sizes for vanadium and uranium, the uranium adsorption capacity is the highest among those competitive ions. All these features indicate that Y‐PAO can serve as a potential adsorbent for selective extraction of uranium from natural seawater.

### 2.3 Uranium Extraction Performance from Natural Seawater

**Figure 5:**
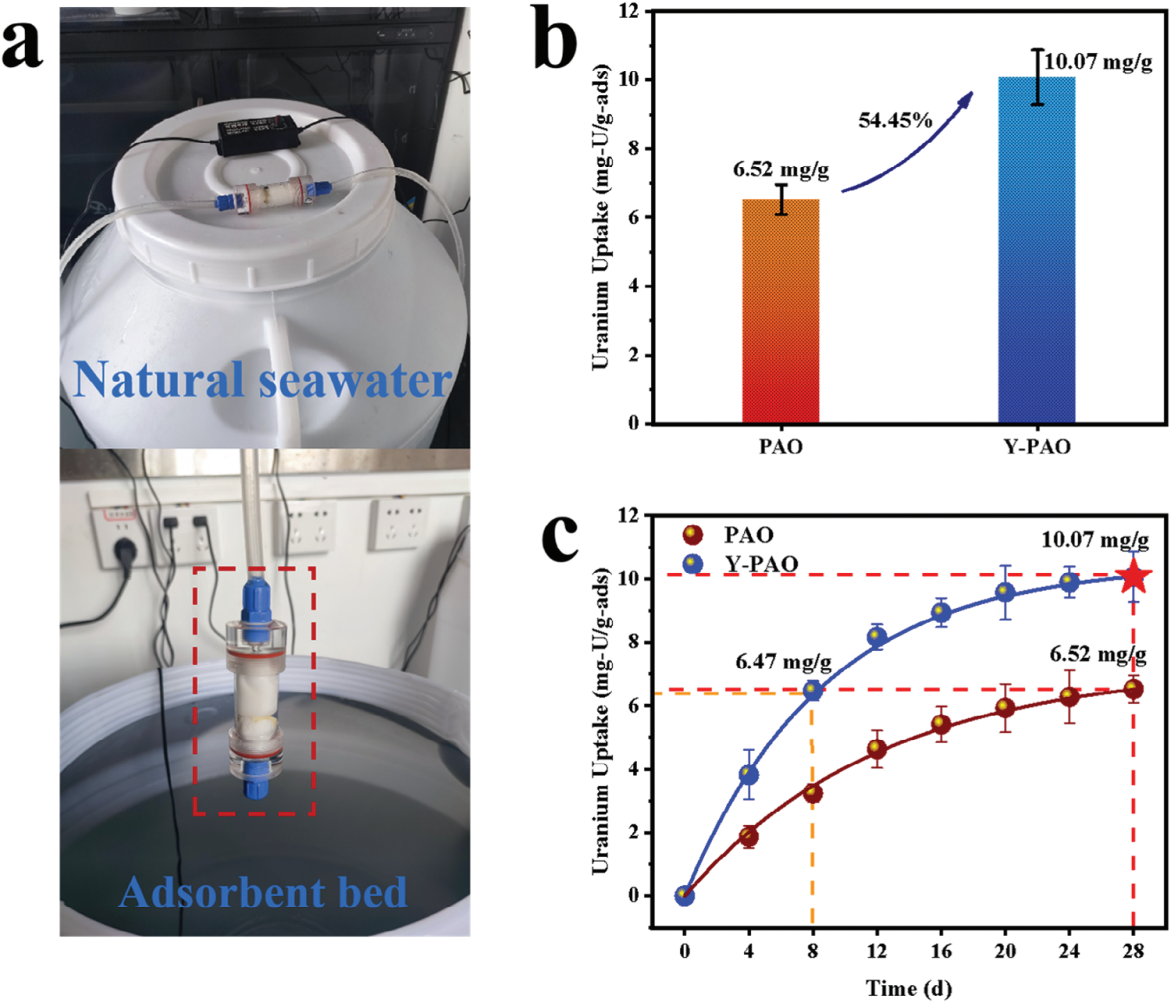
Uranium extraction performance from natural seawater. a) Natural seawater circulation system for uranium extraction. b) Uranium adsorption capacities of PAO and Y‐PAO in natural seawater. c) Uranium adsorption kinetics of PAO and Y‐PAO in natural seawater.

A natural seawater circulation system (**Figure** **5**a) was built in our laboratory to evaluate the uranium extraction performance from natural seawater. After 28 days of contact with circulated natural seawater, the uranium adsorption capacity of Y‐PAO membranes increased by 54.45% compared to that of PAO, reaching up to 10.07 mg‐U/g‐ads (Figure 5b). As observed from the adsorption kinetic data (Figure 5c), the Y‐PAO membrane has not only a higher adsorption capacity, but also a much faster adsorption rate than the PAO membrane. It took Y‐PAO only 8 days to deliver a uranium adsorption capacity close to the equilibrium value of PAO membrane. The fast diffusion channels constructed by hierarchical pores enable Y‐PAO membranes to shorten the adsorption time of uranium, which also mitigate the negative consequences of biofouling and wave impact. After adsorption in natural seawater, the color of the Y‐PAO membrane was darker (Figure S17, Supporting Information). The production cost of Y‐PAO was further estimated in this work. The chemical raw material cost of Y‐PAO used for uranium extraction from seawater is $129.22/kg‐U (Tables S4 and S5, Supporting Information). As the cost of chemical raw materials accounts for approximately 58%–83% of the total cost,^[^ ^36^ ^]^ the total cost of Y‐PAO adsorbent is estimated to be $155.69–$222.79/kg‐U, which is quite close to the current market price of uranium ($123.90/kg‐U as of July 24, 2023). Considering the inevitable rising trend of the uranium price, the Y‐PAO adsorbent will provide tremendous economic benefits in the future.

## 3 Conclusion

In this study, a combination of ice crystal dispersion and yeast‐based biological foaming was employed to prepare a Y‐PAO hydrogel adsorbent with a hierarchical porous structure. The ice crystal dispersion helped preserve the biological activity of yeast in the PAO/DMF system. The hierarchical pores consisted mostly of the large bubble‐like pores generated by yeast respiration as well as the intrinsic micropores of PAO, which facilitated the mass transfer and hence increased the utilization of binding sites. The Y‐PAO membrane exhibited the highest adsorption capacity when the volume ratio of PAO solution and yeast solution was 12.5:1. Moreover, the equilibrium adsorption capacity of Y‐PAO yielded 766.05 mg‐U/g‐Ads, which was 39.90% higher than that of PAO in 8 ppm uranium‐spiked simulated seawater. Meanwhile, Y‐PAO achieved a higher adsorption rate than PAO. In natural seawater, the Y‐PAO membrane delivered a uranium sorption capacity as high as 10.07 mg‐U/g‐Ads, 1.54 times higher than that of the control PAO. Additionally, the Y‐PAO adsorbents demonstrated outstanding reusability and ion selectivity as well as a significant cost advantage. Overall, this work provides a promising strategy to improve the adsorption performance of PAO, thereby facilitating engineering applications in the field of uranium extraction from seawater.

## 4 Experimental Section

### Raw Materials

Instant dry yeast cells (>90 wt%) were acquired from Angel Yeast (China). Analytical‐reagent‐grade glucose (C_6_H_12_O_6_), DMF, sodium carbonate (Na_2_CO_3_), sodium hydroxide (NaOH), and sodium chloride (NaCl) were supplied by Xilong Scientific (China). Uranyl (VI) nitrate hexahydrate (UO_2_(NO_3_)_2_•6H_2_O, 99%) was supplied by Beijing Warwick Chemical (China). Hydroxylamine hydrochloride (NH_2_OH•HCl, analytical reagent grade), general‐reagent‐grade sodium bicarbonate (NaHCO_3_), PAN (99%; average *M*_w_, 150000), and arsenazo III (95%) were obtained from Macklin Biochemical (China). All chemicals were directly used without additional purification.

### Membrane Preparation

A PAO solution was prepared by the oximation of PAN and NH_2_OH•HCl in DMF. First, 13.5 g NH_2_OH•HCl was dissolved in DMF (100 mL), followed by the addition of 10 g Na_2_CO_3_. This solution was agitated for 4 h with a stirrer paddle until the pH value was 7.0. Subsequently, 10 g PAN was added to the solution. The solution was then stirred at 70 °C for 24 h. After stirring, centrifugation was performed at 11,000 rpm for 25 min, yielding a supernatant containing PAO. The proportion of PAO in the DMF solution was approximately 18.31%.

The yeast solution (1 mL) was prepared by mixing instant dry yeast (300 mg) and glucose (100 mg) in purified water. Y‐PAO membranes with different yeast contents were obtained by ice crystal dispersion. The yeast content is represented by the volume ratio between the PAO and yeast solutions, V_PAO_/V_yeast solution_. To prepare the Y‐PAO with V_PAO_/V_yeast solution_ = 12.5, for example, 1 mL yeast solution and 12.5 mL PAO solution were both stored at −18 °C at first. The PAO solution was liquid at −18 °C, whereas the yeast solution was in a solid state. The frozen yeast solution was then pulverized using a mortar and pestle until fine ice crystals were obtained. Afterward, the ice crystals were added to the PAO solution at −18 °C and mixed thoroughly until evenly dispersed. Subsequently, the ice crystal/PAO solution mixture was evenly coated onto a slide to prepare the Y‐PAO membrane, which was finally placed in pure water at 32 °C. Consequently, DMF was replaced by solvent extraction, and the yeast solution thawed and fermented to form pores, yielding the final Y‐PAO membrane. The PAO membrane was prepared similarly but without the yeast solution.

The membranes were subjected to alkali treatment prior to the uranium adsorption experiments. To that end, the samples were placed in a drying oven (70 °C), soaked in a NaOH solution (20 mM) at 60 °C for ≈30 min, and then removed.

### Characterization

The surface microstructures of the membranes were examined by FE‐SEM (S‐4800, Hitachi, Japan). A Bruker Nano XFlash Detector 5030 was used for EDS. Static contact angle testing was conducted using a contact‐angle‐measuring device (OCA 50, DataPhysics Instruments). The elemental valence states and molecular structure were evaluated by XPS (Thermo ESCALAB 250Xi). MIP (AutoPore V 9600) was performed to determine the pore size distributions of PAO and Y‐PAO. A PerkinElmer FTIR spectrometer was used to obtain the FTIR spectra. The uranium concentration was measured with a UV–vis spectrophotometer (UV1800PC) and an inductively coupled‐plasma mass spectrometer (ICP‐MS 7800, Agilent).

### Determination of Optimal Yeast Content and pH

pH experiments were performed with simulated seawater. Deionized water, 25.6322 g NaCl, and 192.97 mg NaHCO_3_ were used to prepare the simulated seawater. The NaCl concentration was 438.607 m, and the NaHCO_3_ concentration was 2.297 mm. To adjust the uranium concentration of this simulated seawater to 8 ppm, it was spiked with 8 mL of uranyl nitrate solution (1000 ppm uranium). pH adjustments were performed with NaOH (10 M) and HCl (1 m) solutions. The adsorption capacity of the prepared adsorbents was tested at pH values of 3.0–9.0. In each experiment, 5 mg (dry weight) of alkali‐treated adsorbent was added to 1000 mL of the simulated seawater with 8 ppm uranium. This mixture was oscillated at a temperature of 35 °C and a speed of 140 rpm. Then, a two‐day (48 h) uranium recovery experiment was conducted in the uranium‐doped simulated seawater. The optimal yeast solution/PAO solution ratio was determined by uniformly mixing the PAO solution (2.5, 5, 7.5, 10, 12.5, 15, 17.5, 20, 22.5, or 25 g) with the yeast solution (1 mL), eventually yielding Y‐PAO films in different proportions through the previously described synthesis process. Uranium adsorption tests were conducted by placing the alkali‐treated Y‐PAO membrane in 1000 mL of simulated seawater solution with a uranium concentration of 8 ppm and pH of 6. Equation (1) was used to calculate the amount of adsorbed uranium:^[^ ^37^ ^]^

(1) $$q_{t}=\frac{\left(C_{0}-C_{t}\right)\times V}{m}\;$$

where *C*_0_ (mg/L) and *C_t_* (mg/L) respectively denote the initial uranium concentration prior to uranium uptake and the instantaneous uranium concentration at contact time *t*, *V* (L) is the volume of uranium‐spiked simulated seawater, *m* signifies the adsorbent dry weight, and *q_t_* (mg‐U/g‐Ads) denotes the amount of adsorbed uranium after time *t*.

### Sorption Kinetics

Simulated seawater solutions with different uranium concentrations (2, 4, 8, 16, 32, 64, and 128 ppm) and a pH value of 6 were used to investigate the adsorption kinetics of the Y‐PAO adsorbent. Briefly, 5 mg of alkali‐treated Y‐PAO was placed in 1000 mL of simulated seawater, and experiments were performed at 140 rpm and 35 °C. The uranium concentration was determined at 4 h intervals. The obtained data were fitted using quasi‐first‐order (Equation (2)) and quasi‐second‐order (Equation (3)) models:^[^ ^38^ ^]^

(2) $$\ln\left(q_{e}-q_{t}\right)={\mathrm{lnq}}_{e}\;-k_{1}t$$

(3) $$\frac{t}{q_{t}}=\frac{1}{k_{2}q_{e}^{2}}\;+\frac{t}{q_{e}}$$

where *t* represents the contact time (min), *k*_1_ and *k*_2_ are rate constants, *q*_e_ (mg L^−1^) denotes the total uranium adsorption capacity of the adsorbent, *q_t_* (mg L^−1^) represents the adsorbed amount of uranium at time *t*, and *t* (min) is the contact time between the adsorbent and the solution.

The equilibrium isotherm data were fitted by the Langmuir (Equation (4)) and Freundlich (Equation (5)) models:

(4) $$\frac{C_{e}}{q_{e}}=\frac{C_{e}}{q_{m}}\;+\frac{1}{k_{3}q_{m}}$$

where *C_e_* is the equilibrium concentration (mg L^−1^), *q_m_* is the saturated adsorption amount (mg g^−1^), and *k*_3_ is an equilibrium constant related to the binding strength (L mg^−1^). *q*_e_ can be determined using the relation:

(5) $$\mathrm{lg}\;q_{e}=\mathrm{lg}\;k_{4}\;+\frac{1}{n}\mathrm{lg}\;C_{e}$$

where *k*_4_ is an approximate indicator of the adsorption capacity and 1/*n* is a function of the strength of adsorption in the adsorption process.

### Membrane Reuse

An eluent containing 1 M Na_2_CO_3_ and 0.1 M H_2_O_2_ was used to elute the uranium from the used adsorbent for approximately 1 h at ≈25 °C. After the elution, the membrane was immersed in pure water for 30 min, subjected to the alkali treatment, and then placed in simulated seawater for an additional uranium adsorption experiment. Equation (6) was used to calculate the elution efficiency^[^ ^3b^ ^]^:

(6) $$EE\;=\frac{C_{el}\times V_{el}}{\left(C_{i}-C_{f}\right)\times V_{u}}\;\times 100\%$$

where *EE* (%) is the elution efficiency, *C*_el_ (mg L^−1^) represents the uranium concentration of the eluent, *V*_el_ (L) represents the eluent volume, *C*_i_ and *C*_f_ respectively denote the initial and final concentrations of uranium, and *V_u_* (L) denotes the volume of the simulated seawater spiked with uranium (L).

### Adsorption Selectivity

The ability of the adsorbent to extract uranium under the interfering effects of unrelated ions was evaluated by performing an ion selectivity experiment. Simulated seawater was made from natural seawater with the addition of various metal salts (the salts of U, V, Mn, Cr, Fe, Ni, Co, Pb, Mo, and Cu). All metal ion concentrations were 100 times higher than their normal concentrations in natural seawater (Table S6, Supporting Information). We used 1000 mL of this simulated seawater with 5 mg (dry weight) of the alkaline‐treated adsorbent. Uranium extraction was performed with oscillation at 140 rpm for 24 h at 35 °C. The uranium adsorption selectivities of PAO and Y‐PAO and their selectivities for the other unrelated ions were evaluated using the adsorption capacities for the diverse ions estimated by ICP‐MS.

### Uranium Collection in Natural Seawater

Natural seawater (uranium concentration, ≈3.3 ppb; 200 L) was obtained near the west coast of Haikou City, Hainan Province, China, in the South China Sea. This seawater was used to evaluate the applicability of the prepared adsorbents in a real environment. The seawater was injected into a circulating water system, and the alkali‐treated adsorbent (10 mg) was attached to a sponge in the drying tube of the circulating water system (Figure 5a). The tests were conducted for 28 days, and sampling was conducted every four days. ICP‐MS was employed to measure the change in uranium concentration in the seawater.

## Conflict of Interest

The authors declare no conflict of interest.

## Supporting information

Supporting Information

## Data Availability

The data that support the findings of this study are available in the supplementary material of this article.
