# Supplementary material for: Yeast‐Raised Polyamidoxime Hydrogel Prepared by Ice Crystal Dispersion for Efficient Uranium Extraction from Seawater
Source: Adv Sci (Weinh). 2024 Feb 13;11(17):2306534. doi: 10.1002/advs.202306534 (PMC11077670; doi:10.1002/advs.202306534)
Supplement: Supplementary file 1 — Supporting Information [file ADVS-11-2306534-s001.pdf]

ADVANCED SCIENCE
Open Access

Supporting Information

for *Adv. Sci.*, DOI 10.1002/advs.202306534

Yeast-Raised Polyamidoxime Hydrogel Prepared by Ice Crystal Dispersion for Efficient Uranium Extraction from Seawater

Hui Wang, Weikun Yao, Yihui Yuan, Se Shi, Tao Liu and Ning Wang**

Supporting Information

Yeast-raised polyamidoxime hydrogel prepared by ice crystal dispersion for efficient uranium extraction from seawater

Hui Wang[1,2], Weikun Yao[1,2], Yihui Yuan[1], Se Shi[1], Tao Liu*[1], Ning Wang*[1]

[1] State Key Laboratory of Marine Resource Utilization in South China Sea

Hainan University, Haikou 570228, China

* Corresponding authors. *E-mail addresses*: wangn02@foxmail.com (Ning Wang)

liutao565@126.com (Tao Liu)

[2] These authors contributed equally to this work.

Table of Contents

Figure S1 | High-resolution XPS spectra for Y-PAO membrane in C 1s area. ··················· 1

Figure S2 | SEM images of yeast cell. ··· 2

Figure S3 | Mercury intrusion-extrusion curves of PAO and Y-PAO membranes. ··············· 3

Figure S4 | Optical micrographs (×60) of PAO and Y-PAO membranes. ························ 4

Figure S5 | The weight of the square PAO and Y-PAO membranes (1 cm×1 cm×100 μm). ···· 5

Figure S6 | Tensile stresses of Y-PAO membranes with different yeast solution contents. ····· 6

Figure S7 | Thicknesses of PAO and Y-PAO membranes measured by a digital micrometer. ·· 7

Figure S8 | Volume changes for PAO and Y-PAO membranes before and after alkali treatment. ··· 8

Figure S9 | SEM images of Y-PAO membranes with different volume ratio of PAO and yeast. ··· 9

Figure S10 | Uranium adsorption performance of PAO and Y-PAO membranes in U-spiked simulated seawater (C_0, 2 ppm; V, 1000 mL; M_{ads}, 5 mg). ······································ 10

Figure S11 | Uranium adsorption performance of PAO and Y-PAO membranes in U-spiked simulated seawater (C_0, 4 ppm; V, 1000 mL; Mads, 5 mg). ······································ 11

Figure S12 | Uranium adsorption performance of PAO and Y-PAO membranes in U-spiked simulated seawater (C_0, 16 ppm; V, 1000 mL; Mads, 5 mg). ···································· 12

Figure S13 | EDS analysis of uranium-loaded Y-PAO. ·· 13

Figure S14 | High-resolution XPS spectra of O 1s for Y-PAO before and after uranium adsorption. ··· 14

Figure S15 | High-resolution XPS spectra of N 1s for Y-PAO after uranium adsorption.······15

Figure S16 | Chemical coordination mechanism between amidoxime group and UO_2^{2+}.······15

Figure S17 | Digital photographs of Y-PAO membrane before and after uranium adsorption in nature seawater. ···17

Table S1 | Fitting parameters of adsorption kinetics data based on pseudo-second-order and pseudo-first-order models. ···18

Table S2 | Uranium adsorption capacities of PAO and Y-PAO membranes at different times. 19

Table S3 | Fitting parameters of equilibrium adsorption isotherms of Y-PAO based on Langmuir and Freundlich models. ···20

Table S4 | Uranium adsorption capacity of Y-PAO adsorbent at different cycle numbers in natural seawater. ···21

Table S5| Economic cost of industrial raw materials for 1 kg uranium production. ···········22

Table S6 | Concentration of uranium and competitive metal ions in natural seawater and metal-ion-spiked seawater. ···23

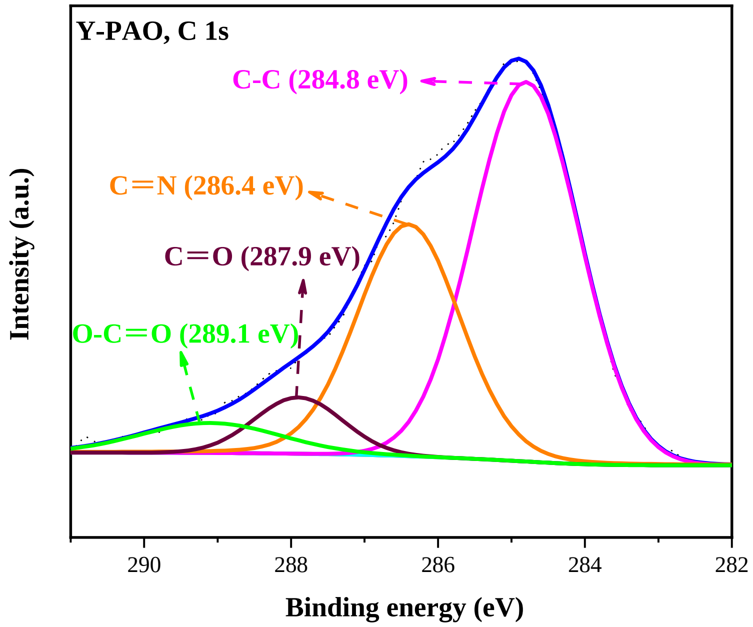

Figure S1. High-resolution XPS spectra for Y-PAO membrane in C 1s area.

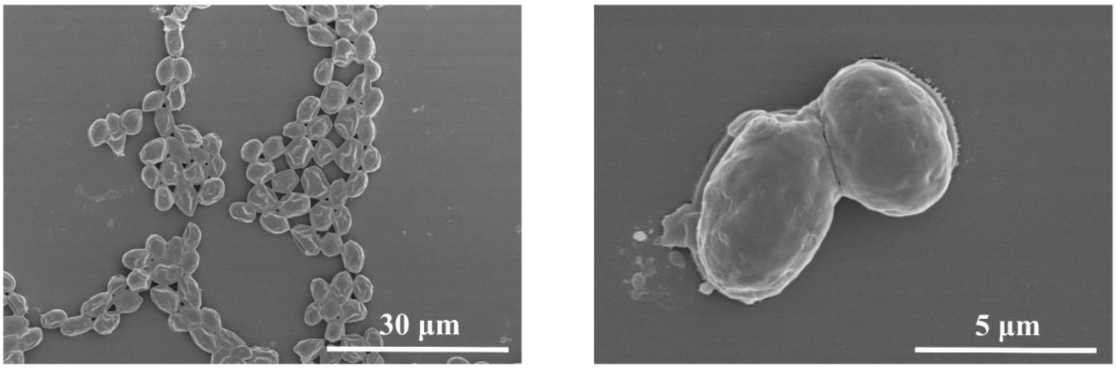

Figure S2. SEM images of yeast cell.

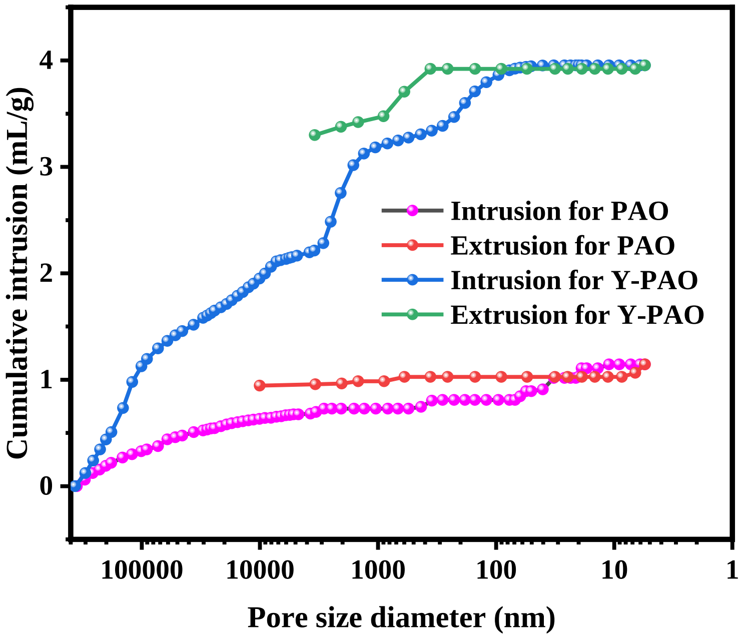

Figure S3. Mercury intrusion-extrusion curves of PAO and Y-PAO membranes.

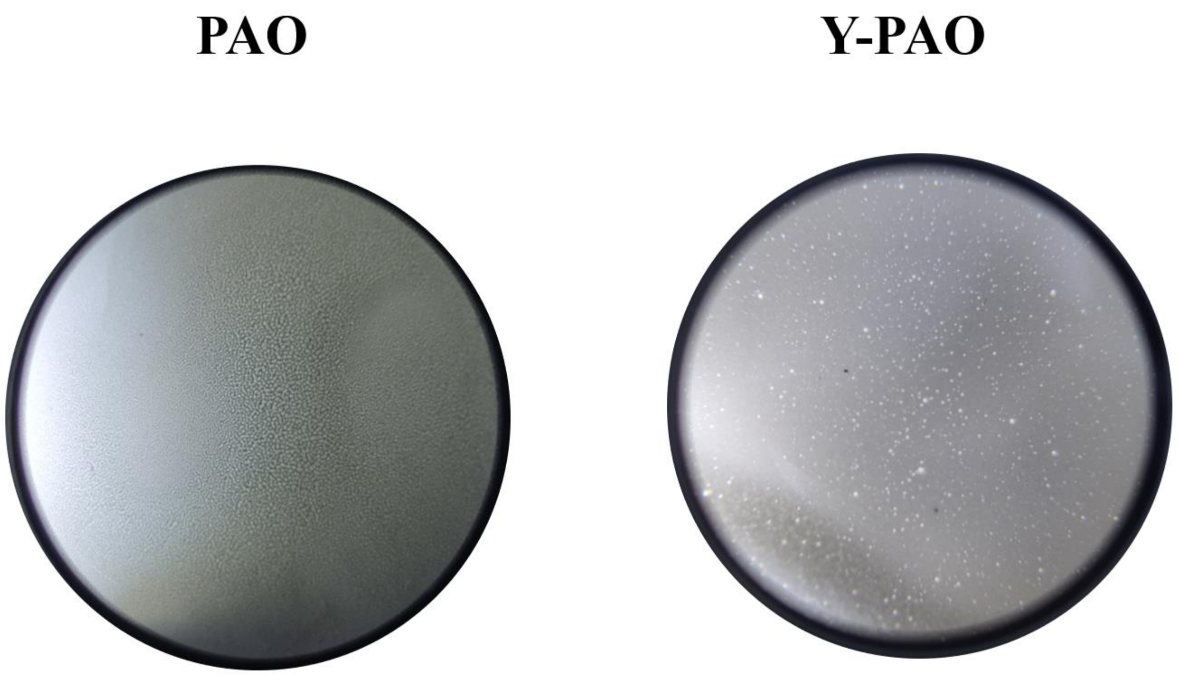

Figure S4. Optical micrographs (×60) of PAO and Y-PAO membranes.

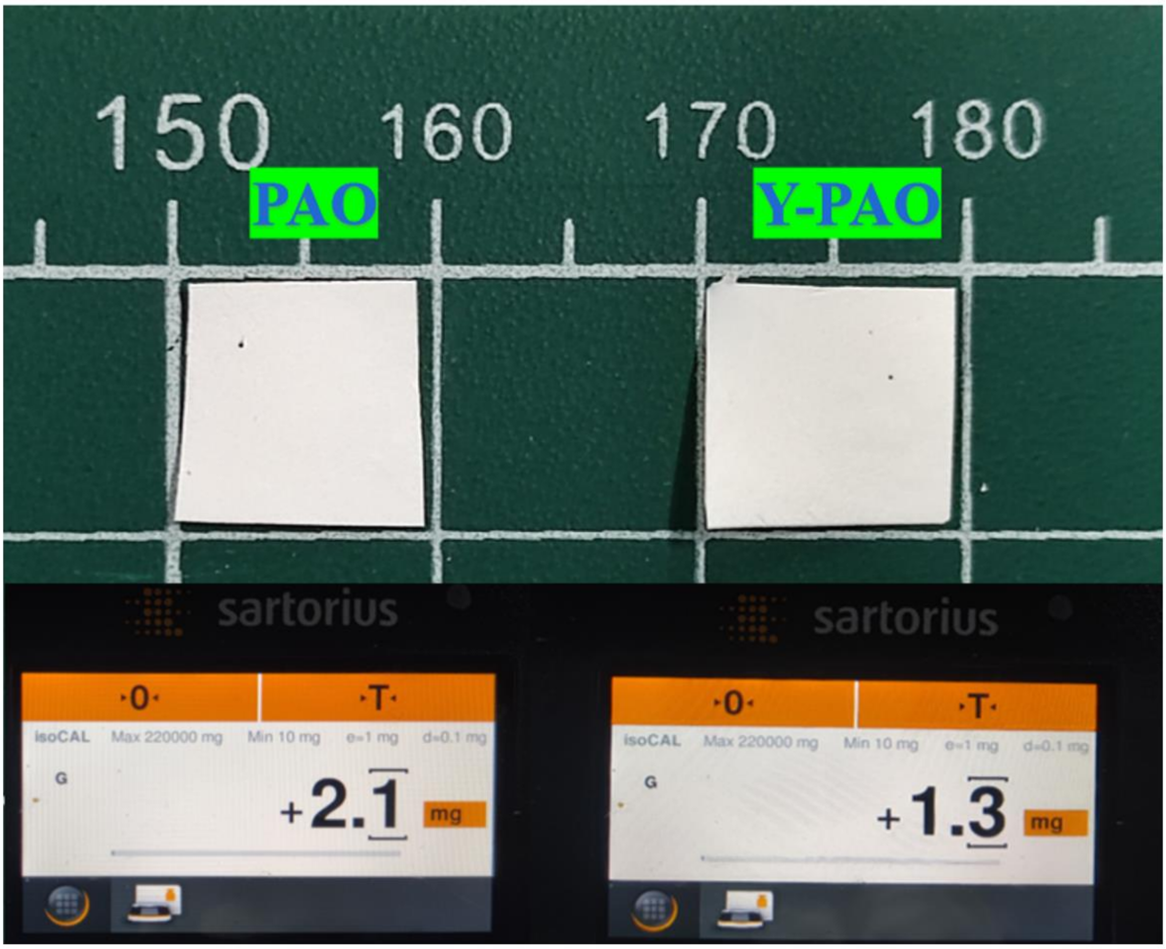

Figure S5. The weight of the square PAO and Y-PAO membranes (1 cm×1 cm×100 μm).

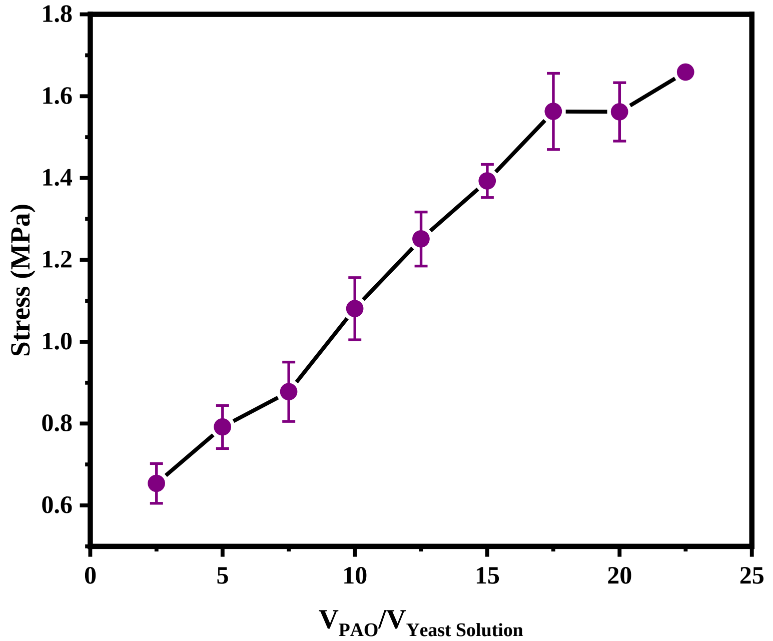

Figure S6. Tensile stresses of Y-PAO membranes with different yeast contents.

PAO **Y-PAO**

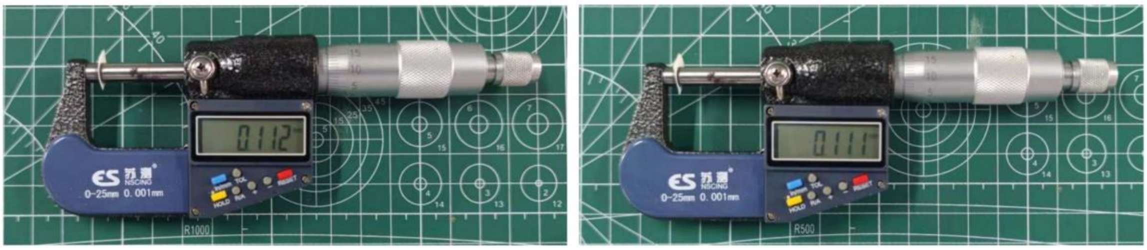

Figure S7. Thicknesses of PAO and Y-PAO membranes measured by a digital micrometer.

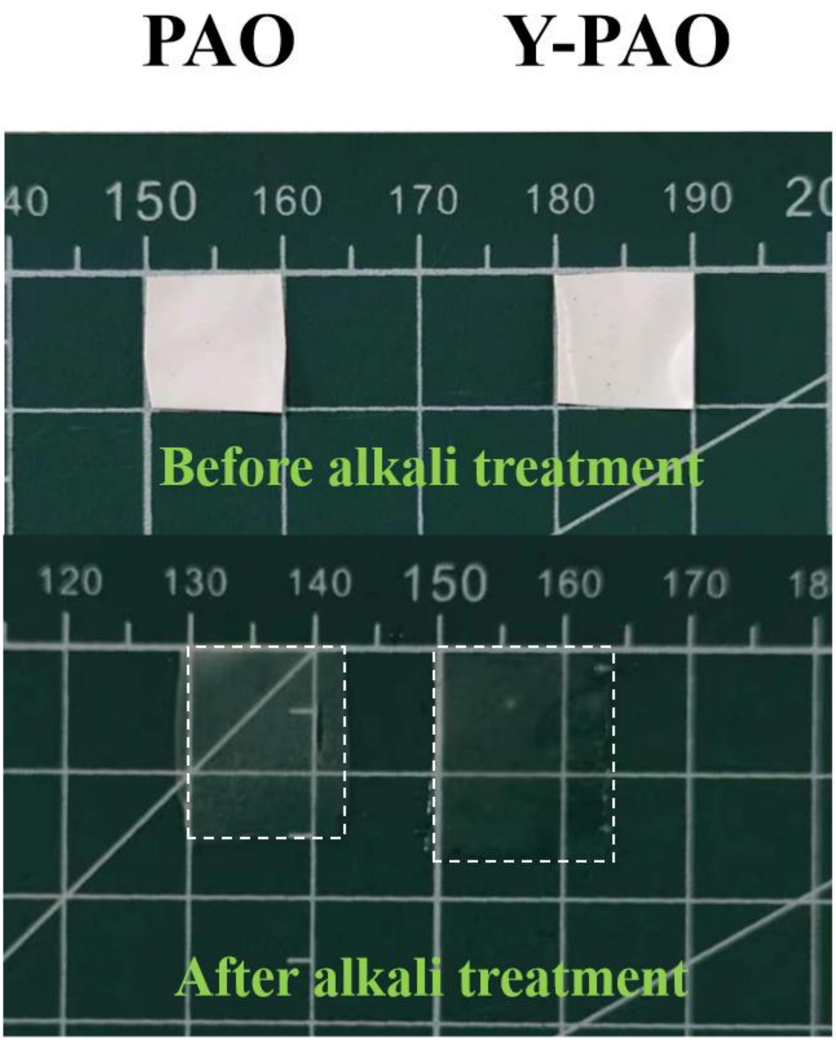

Figure S8. Volume changes for PAO and Y-PAO membranes before and after alkali treatment.

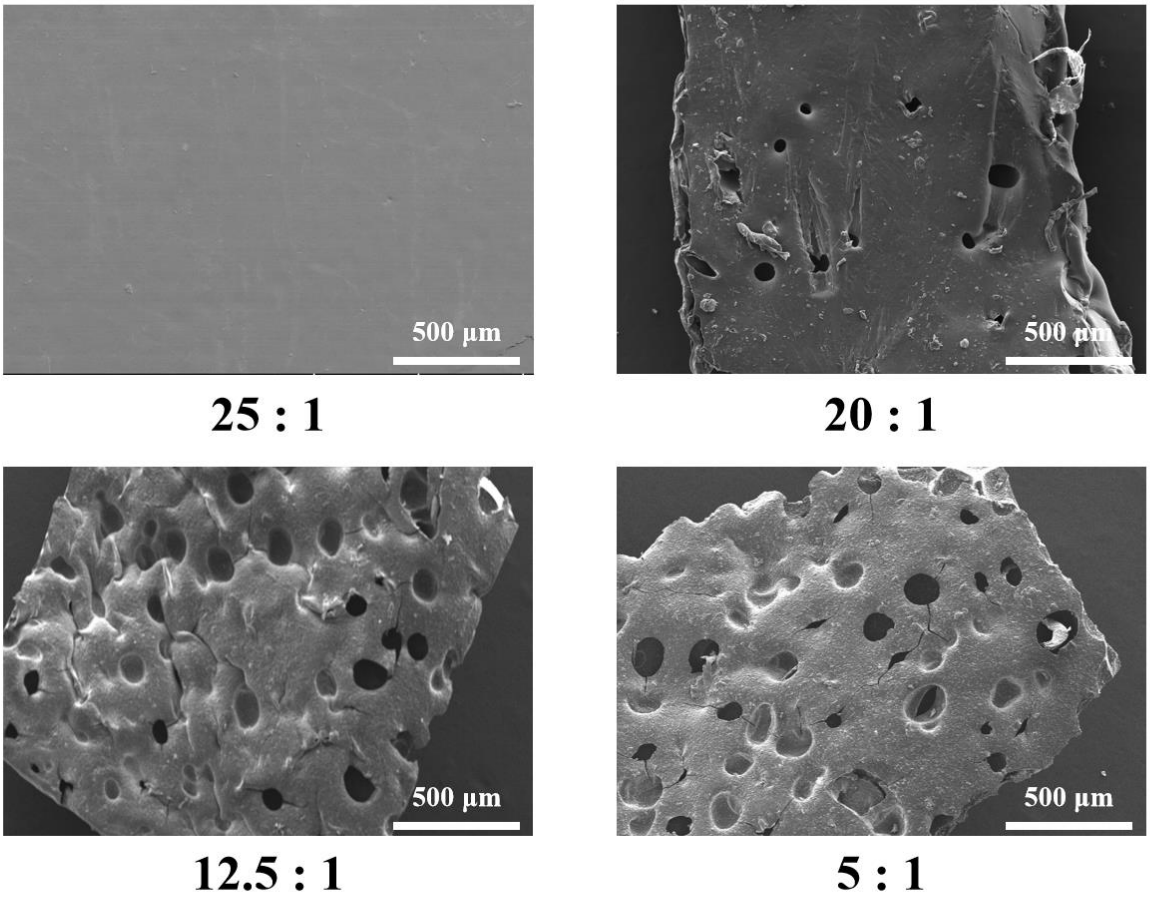

Figure S9. SEM images of Y-PAO membranes with different solution volume ratio of PAO and yeast.

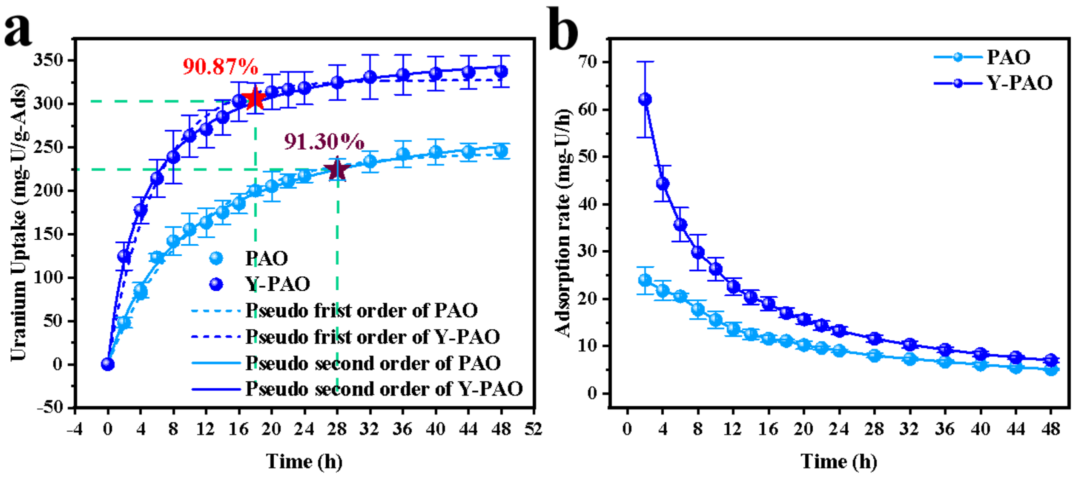

Figure S10. Uranium adsorption performance of PAO and Y-PAO membranes in U-spiked simulated seawater (C_0, 2 ppm; V, 1000 mL; M_{ads}, 5 mg). a) Uranium adsorption kinetics data and b) Uranium adsorption rate.

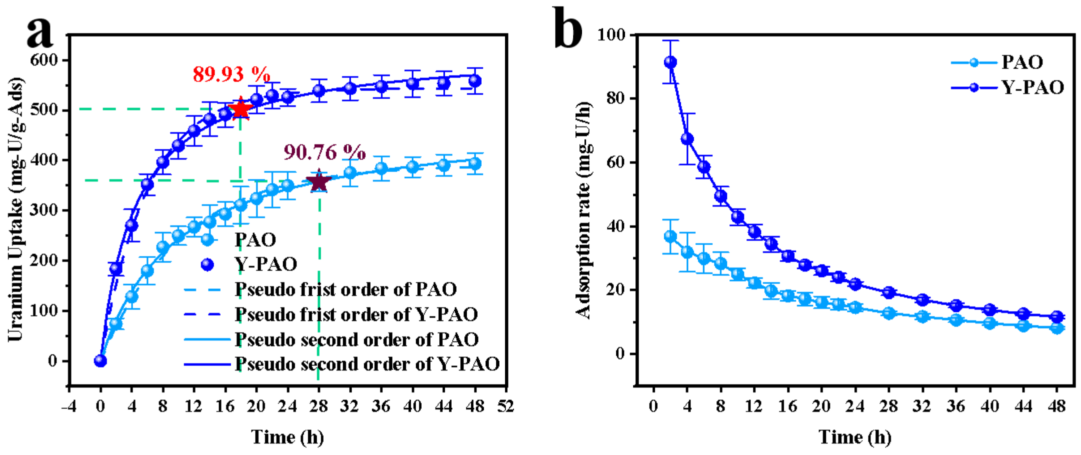

Figure S11. Uranium adsorption performance of PAO and Y-PAO in U-spiked simulated seawater (C_0, 4 ppm; V, 1000 mL; M_{ads}, 5 mg). a) Uranium adsorption kinetics data and b) Uranium adsorption rate.

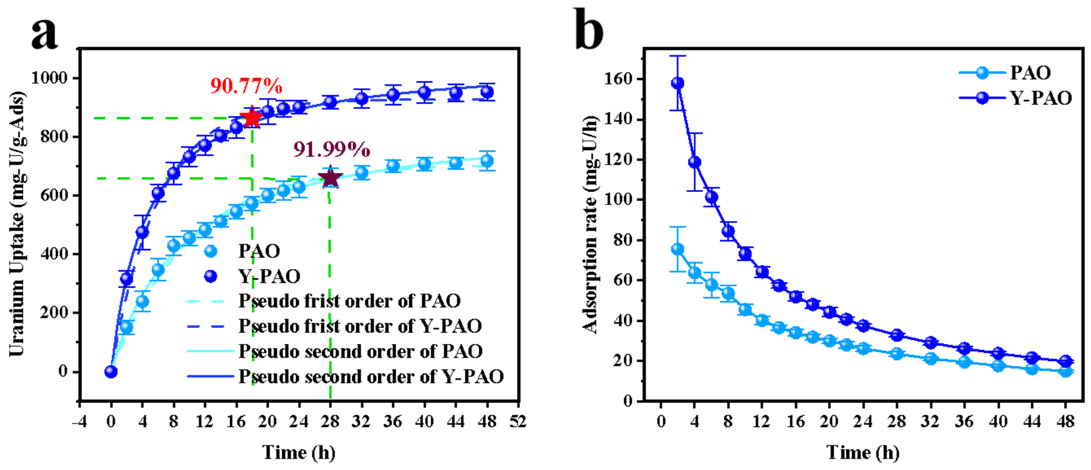

Figure S12. Uranium adsorption performance of PAO and Y-PAO in U-spiked simulated seawater (C_0, 16 ppm; V, 1000 mL; M_{ads}, 5 mg). a) Uranium adsorption kinetics data and b) Uranium adsorption rate.

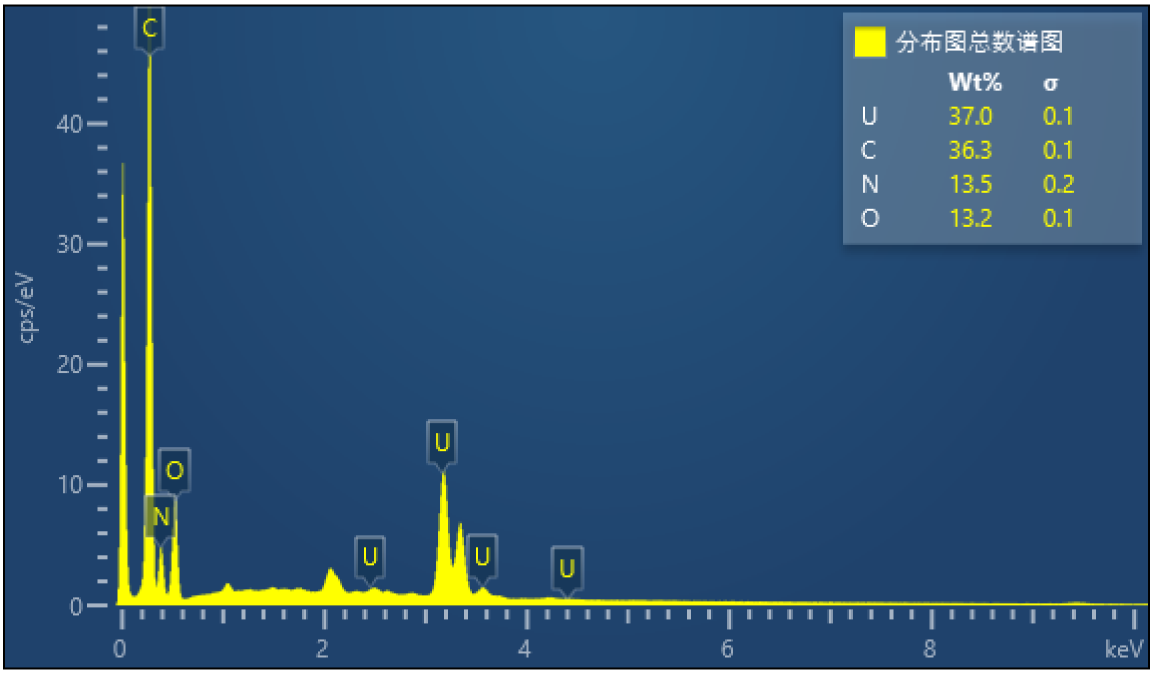

Figure S13. EDS analysis of uranium-loaded Y-PAO.

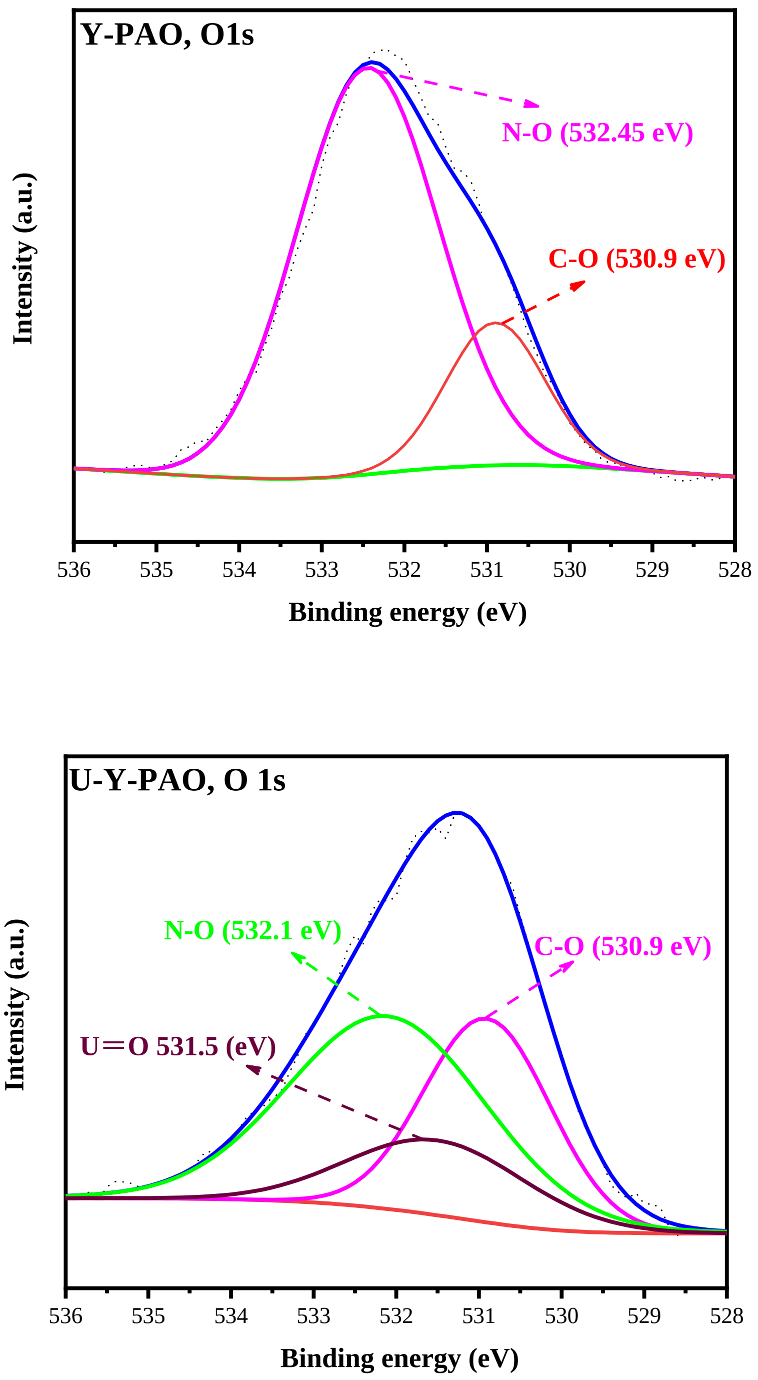

Figure S14. High-resolution XPS spectra of O 1s for Y-PAO before and after uranium adsorption.

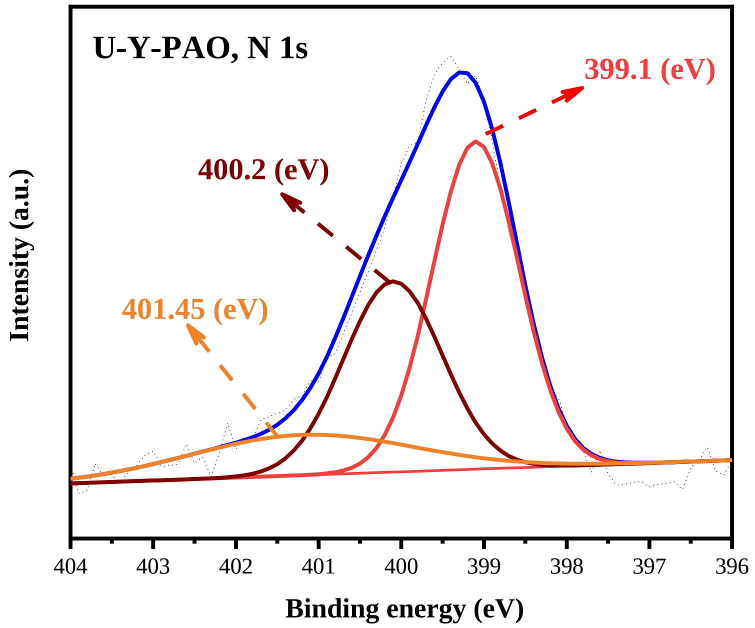

Figure S15. High-resolution XPS spectra of N 1s for Y-PAO after uranium adsorption.

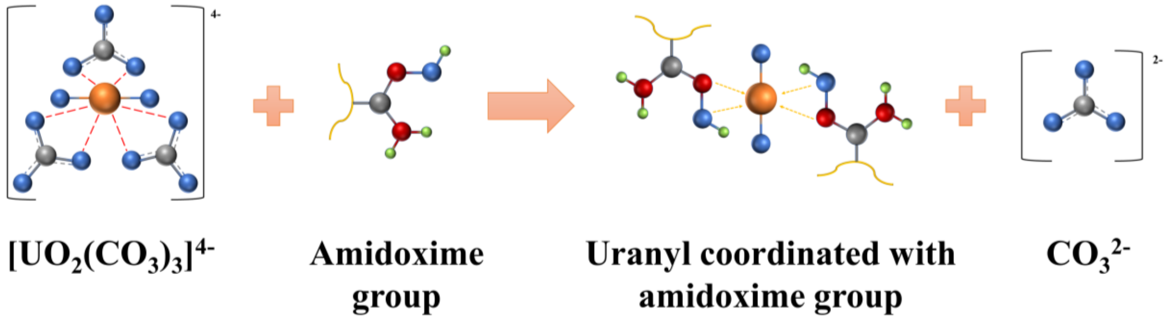

Figure S16. Chemical coordination mechanism between amidoxime group and UO_2^{2+}.

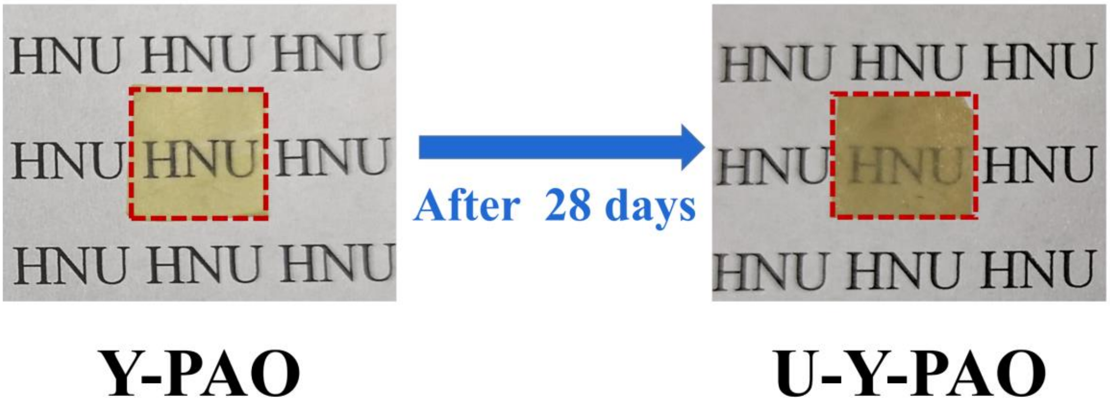

Figure S17. Digital photographs of Y-PAO membranes before and after uranium adsorption in nature seawater.

Table S1. Fitting parameters of adsorption kinetics data based on pseudo-second-order and pseudo-first-order models.

	C_o (ppm)	Pseudo-second-order		Pseudo-first-order	
		R^2	q_e (mg g^{-1})	R^2	q_e (mg g^{-1})
PAO	2	0.99702	301.10	0.99276	244.05
	4	0.99705	484.91	0.99475	390.73
	8	0.99783	663.69	0.99463	540.78
	16	0.99749	869.86	0.99276	707.57
Y-PAO	2	0.99784	373.38	0.98606	327.50
	4	0.99635	624.82	0.99446	543.70
	8	0.99754	856.85	0.99204	747.96
	16	0.99825	1066.08	0.99125	928.14

Table S2. Uranium adsorption capacities of PAO and Y-PAO membranes at different times.

	PAO				Y-PAO			
	**2 ppm**	**4 ppm**	**8 ppm**	**16ppm**	**2 ppm**	**4 ppm**	**8 ppm**	**16 ppm**
0	0.00	0.00	0.00	0.00	0.00	0.00	0.00	0.00
2	47.90	73.70	109.75	151.08	124.40	182.94	264.88	315.93
4	86.98	127.77	186.50	239.04	177.61	269.70	385.71	474.96
6	123.17	179.64	269.32	346.98	214.11	351.82	479.52	608.21
8	141.95	226.78	322.68	428.82	238.74	396.03	550.20	675.27
10	155.52	249.56	354.77	455.24	263.10	429.25	594.37	731.68
12	163.31	267.04	375.18	482.72	270.73	458.83	622.01	770.91
14	175.13	276.68	398.78	511.83	284.66	481.83	657.39	803.58
16	185.03	292.50	418.64	545.48	303.05	490.88	680.66	831.07
18	200.15	310.60	439.25	573.20	306.60	502.04	698.28	864.31
20	205.01	323.90	461.22	600.39	313.71	521.25	714.69	885.71
22	211.16	341.37	477.29	616.78	316.62	529.06	722.47	895.02
24	216.86	349.28	490.14	629.05	318.30	525.49	726.04	900.17
28	224.37	357.15	503.96	660.38	324.68	538.78	743.05	917.85
32	233.60	375.13	517.20	677.63	330.89	543.08	754.40	929.90
36	242.22	383.89	534.57	700.89	333.59	547.15	756.31	942.85
40	244.49	386.47	540.88	707.56	334.73	552.51	760.78	951.55
44	244.59	389.89	544.83	710.55	336.49	553.43	763.41	949.79
48	245.76	393.51	547.58	717.90	337.40	558.26	766.05	952.25

Table S3. Fitting parameters of equilibrium adsorption isotherms of Y-PAO based on Langmuir and Freundlich models.

Langmuir model			Freundlich model		
R^2	k_3 (L mg^{-1})	q_e (mg g^{-1})	R^2	k_4 (mg g^{-1})	n
0.99834	0.12405	1182.83	0.98991	1152.32	1.58227

Table S4. Uranium adsorption capacity of Y-PAO adsorbent at different cycle numbers in natural seawater.

Recycles	Adsorption efficiency (%)	Adsorption capacity (mg g^{-1})	Elution efficiency (%)	Elution Capacity (mg g^{-1})
1	100	10.07	96.12	9.68
2	94.37	9.50	92.48	8.79
3	89.53	9.02	89.36	8.06
4	83.96	8.45	86.70	7.33
5	78.81	7.94	83.74	6.65
**Total**	/	/	/	**40.51 mg g^{-1}**

The adsorption capacity is estimated based on 5-cycle reusability test of Y-PAO in U-spiked simulated seawater.

Table S5. Economic cost of industrial raw materials for 1 kg uranium production.

Raw material	Price per kg	Quantity	Total price
$NH_2OH·HCl$	$2.09	14.15 kg	$29.57
PAN	$2.17	10.47 kg	$22.72
Na_2CO_3	$0.42	10.47 kg	$4.40
DMF	$0.70	99.36 kg	$69.55
Yeast	$1.15	2.31 kg	$2.66
Glu	$0.42	0.76 kg	$0.32
**Total**	/	/	**$129.22**

Table S6. Concentration of uranium and competitive metal ions in natural seawater and metal-ion-spiked seawater.

Metal	Natural seawater (ppb)	Metal ion-spiked seawater (ppb)
**U**	3.3	330
**V**	1.5	150
**Mn**	0.2	20
**Cr**	0.3	30
**Fe**	1.0	100
**Ni**	1.0	100
**Co**	0.05	5
**Pb**	0.03	3
**Mo**	10.6	10.6
**Cu**	0.6	60
